# Comment on Dyńka et al. The Ketogenic Diet and Cardiovascular Diseases. *Nutrients* 2023, *15*, 3368

**DOI:** 10.3390/nu15204311

**Published:** 2023-10-10

**Authors:** Rami Salim Najjar

**Affiliations:** Institute for Biomedical Sciences, Georgia State University, Atlanta, GA 30303, USA; rnajjar1@gsu.edu

The recent review by Dyńka et al. [1] concludes that consuming a ketogenic diet (KGD) is a favorable strategy for cardiovascular disease (CVD) prevention. However, this conclusion is not supported by the evidence presented by the authors and also contradicts an expert review panel of the American Heart Association (AHA) which ranked KGDs as the worst diets for preventing CVDs [2]. Some major points are highlighted below:

(I) The authors call into question the link between elevated low-density lipoprotein cholesterol (LDL-C) and CVD, as they state that association is not causation. However, the AHA presidential panel ranked the link between LDL-C and CVD to reach the level of evidence to meet causality, and this conclusion was reached unanimously by three independent guideline committees based on “Level A, Strong” evidence [3]. I suspect that these lines of evidence detailed by AHA were not considered when making such a paradigm-shifting claim.

(II) Despite the authors’ prior dismissal of the link between LDL-C and CVD, they contradictorily proceed to argue that a KGD is favorable in preventing CVD by reducing LDL-C. In any case, the authors cite mostly trials in which diets were designed to be hypocaloric to draw this conclusion. The confounding effects of weight loss were not considered when evaluating the lipid-mediating effects of a KGD. For example, carefully designed metabolic ward studies have revealed that ad libitum intake or weight-maintaining KGDs significantly increases LDL-C and all subfractions [4,5]. It has been known for decades that increasing saturated fat consumption, inherent in the KGD, will blunt hepatic LDL receptors, resulting in a rise in LDL-C [6].

(III) The authors claim that a KGD is more effective in reducing body weight compared to other diets. Ignoring the fact that most of the trials examined were designed to be hypocaloric, it has been demonstrated in a convincing metabolic ward study that calorie-for-calorie, dietary fat restriction reduces fat mass to a greater extent than carbohydrate restriction [7]. In fact, body weight may reduce to a greater extent with carbohydrate restriction due to reductions in lean mass.

(IV) The authors claim that a KGD will favorably impact blood glucose, however, a KGD will also increase fasting free fatty acids (FFAs) [4,5]. Elevated FFAs impair insulin signaling, resulting in insulin resistance, a well-understood molecular concept [8]. While reducing dietary carbohydrates on a KGD inherently reduces fasting glucose, a carbohydrate challenge on a KGD demonstrates overt insulin resistance [4]. Further, elevated FFAs can impair endothelial function [9], a physiological consequence in contradiction to authors’ conclusions that a KGD improves endothelial function. Indeed, consuming low-carbohydrate diets result in reduced endothelial function overall [10].

The misleading conclusions made by authors will send the wrong public health message, dangerously promoting the use of KGDs in treating CVDs, when, in fact, a KGD likely exacerbates CVD. 
